# The Inhibition of RANKL-Induced Osteoclastogenesis through the Suppression of p38 Signaling Pathway by Naringenin and Attenuation of Titanium-Particle-Induced Osteolysis

**DOI:** 10.3390/ijms151221913

**Published:** 2014-11-28

**Authors:** Wengang Wang, Chuanlong Wu, Bo Tian, Xuqiang Liu, Zanjing Zhai, Xinhua Qu, Chuan Jiang, Zhengxiao Ouyang, Yuanqing Mao, Tingting Tang, An Qin, Zhenan Zhu

**Affiliations:** 1 Shanghai Key Laboratory of Orthopedic Implants, Department of Orthopedics, Shanghai Ninth People’s Hospital, Shanghai Jiao Tong University School of Medicine, Shanghai 200011, China; 2 Department of Orthopedics, Hunan Provincial Tumor Hospital and Tumor Hospital of Xiangya School of Medicine, Central South University, Changsha 410013, China

**Keywords:** naringenin, osteolysis, osteoclast, p38 signaling, NFATc1

## Abstract

The aim of this study was to assess the effect of naringenin on osteoclastogenesis and titanium particle-induced osteolysis. Osteolysis from wear-induced particles and aseptic loosening are the most frequent late complications of total joint arthroplasty leading to revision of the prosthesis. Osteolysis during aseptic loosening is most likely due to increased bone resorption by osteoclasts. Through *in vitro* studies, we demonstrated that naringenin, a naturally occurring flavanone in grapefruit and tomatoes, exerts potent inhibitory effects on the ligand of the receptor activator of nuclear factor-κB (RANKL)-induced osteoclastogenesis and revealed that the mechanism of action of naringenin, which inhibited osteoclastogenesis by suppression of the p38 signaling pathway. Through *in vivo* studies, we proved that naringenin attenuated titanium particle-induced osteolysis in a mouse calvarial model. In general, we demonstrated that naringenin inhibited osteoclastogenesis via suppression of p38 signaling *in vitro* and attenuated titanium particle-induced osteolysis *in vivo*. This study also suggested that naringenin has significant potential for the treatment of osteolysis-related diseases caused by excessive osteoclast formation and activity.

## 1. Introduction

The goal of total joint arthroplasty (TJA) is to relieve joint pain and improve function, and this procedure can have a significant impact on health-related quality of life [1]. Currently, it is estimated that about 3.5 million TJAs are performed worldwide each year. In the USA, the demand for hip revision procedures was projected to double by the year 2026, while the demand for knee revisions was expected to double by 2015 [2]. Although much effort has been made to improve the efficacy of TJA, particle-induced periprosthetic osteolysis and subsequent aseptic loosening continue to be major causes of arthroplasty failure [3]. Studies suggest that osteolysis at the periprosthetic site results from excessive osteoclast activation, which is initiated by activation of the receptor activator of nuclear factor-κB (RANK) and RANK ligand (RANKL) signaling pathways [4,5]. RANKL signaling triggers osteoclast differentiation and is an important target for treating pathological bone loss [6]. Docking of RANKL to its receptor, RANK, rapidly activates MAPKs (mitogen-activated protein kinases) such as p38, extracellular signal-regulated kinase (ERK) and c-Jun *N*-terminal kinase (JNK). These MAPKs are essential for the differentiation, survival and activation of osteoclasts [7,8].

Recently, the involvement of the p38 pathway in osteoclast differentiation and osteolysis has been an area of active research. Activation of p38 signaling induced osteolytic bone lesions by inhibiting osteoblastogenesis and enhancing osteoclastogenesis [9]. A p38 inhibitor blocked RANKL-induced osteoclastogenesis [10] and osteolysis due to wear-induced particles [11]. Mice deficient in the *p38 MAPK* gene showed significantly less osteoclastogenesis than control mice. p38 signaling could be one of the main factors regulating the inflammatory process in wear particle-induced osteolysis [12]. Taken together, these data indicate that although other pathways such as NF-κB and JNK are essential for RANKL-induced osteoclastogenesis, p38 MAPK-mediated signals are crucial in osteoclast differentiation and wear debris-induced osteolysis. The inhibition of p38 MAPK activation has demonstrated clinical efficacy on bone destruction in experimental arthritis models. Several clinical trials are testing the efficacy of these compounds in human disease [13,14,15].

**Figure 1:**
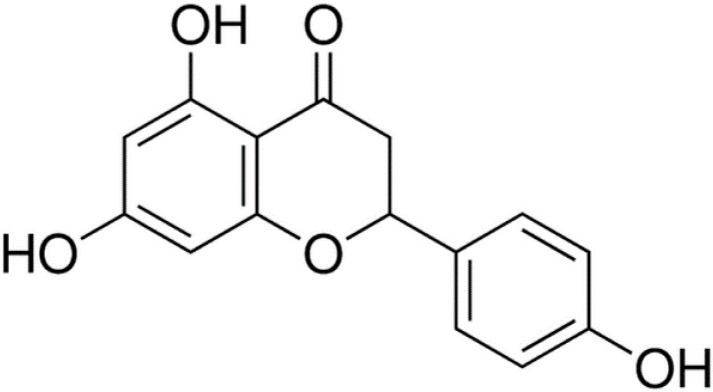
Chemical structure of naringenin (NAR) with a molecular formula of C_15_H_12_O_5_ and a molecular weight of 272.25 g/mol.

Naringenin (4,5,7-trihydroxyflavanone, NAR, Figure 1), a naturally occurring flavanone in grapefruit and tomatoes [16,17], has been demonstrated to elicit a wide range of pharmacological and biological effects, including anti-oxidant [18] anti-inflammatory [19] and anti-neoplastic activities [20]. Recently, some unexpected properties of NAR have generated a great deal of attention. NAR was found to inhibit the formation of osteoclasts and their resorptive activity in rabbit bone marrow cells [21]. Surprisingly, NAR was also shown to inhibit human osteoclastogenesis and osteoclastic bone resorption [22]. In addition, NAR was reported to possess osteogenic effects [23,24,25]. However, it remains unclear that the mechanism of action of its inhibition of osteoclastogenesis *in vitro* and whether it can prevent osteolytic diseases *in vivo*.

Given the importance of the p38 signaling pathway in osteoclast formation and the effects of NAR on p38 suppression in other cell types [26,27,28,29,30], we hypothesized that NAR might inhibit osteoclastogenesis via suppression of p38 signaling. To test this hypothesis, we performed a series of assays *in vitro*. To investigate NAR’s effect *in vivo*, we established a mouse model of titanium particle-induced osteolysis and then used micro-computed tomography (micro-CT) scanning and histomorphometric analysis to examine calvarial samples.

## 2. Results

### 2.1. NAR Cytotoxicity and Effect of NAR on Osteoclastogenesis

**Figure 2:**
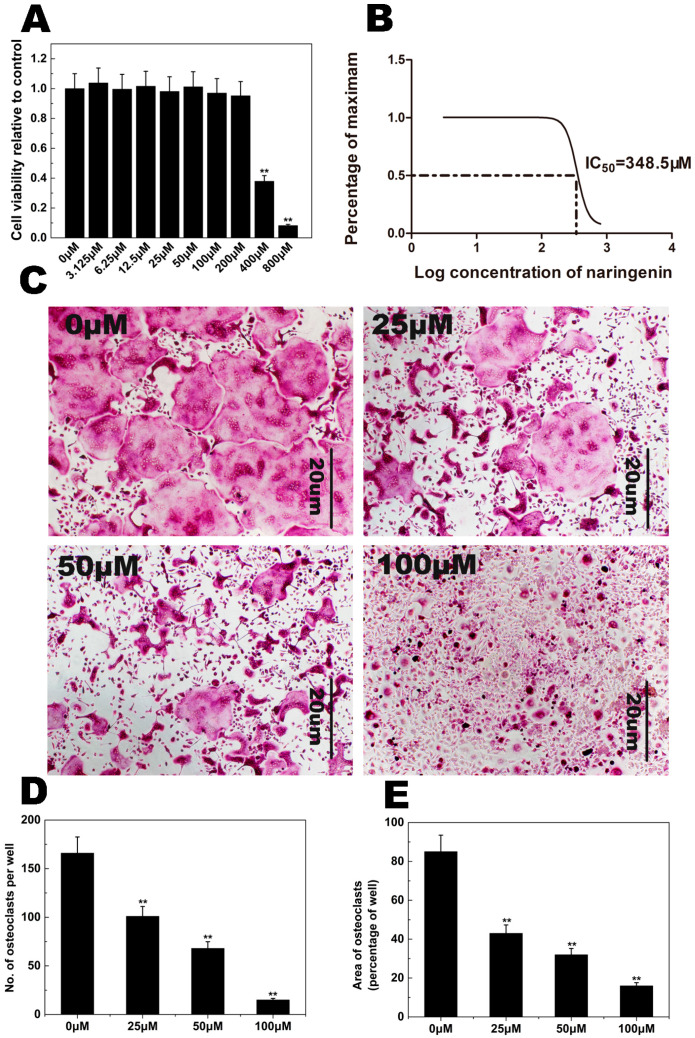
Naringenin (NAR) inhibited receptor activator of the nuclear factor-κB ligand (RANKL)-induced osteoclastogenesis. (**A**) Bone marrow macrophages (BMMs) were cultured in complete α-MEM medium containing the indicated concentrations of NAR for 48 h. Cell viability, relative to control, was measured by CCK-8 assay; (**B**) The inhibition of BMM viability was analyzed using GraphPad Prism software (GraphPad Software, La Jolla, CA, USA), and the half-maximal inhibitory concentration (IC_50_) was determined to be 348.5 μM; (**C**) BMMs were treated with different concentrations of NAR followed by 30 ng/mL macrophage colony-stimulating factor (M-CSF) and 50 ng/mL RANKL for 5–7 days. Cells were then fixed with 4% paraformaldehyde and stained for tartrate-resistant acid phosphatase (TRAP); (**D**) The number of TRAP-positive cells; (**E**) The area occupied by TRAP-positive cells. ** *p* < 0.01.

CCK-8 cell viability assays were performed to examine the potential cytotoxicity of NAR. Our results showed that NAR cytotoxicity was concentration-dependent (Figure 2A,B). These assays indicated that the maximum concentration used in our subsequent studies (200 μM) showed no cytotoxic effects in BMMs. Therefore, the NAR concentrations used in subsequent experiments were considered non-cytotoxic.

To investigate the effect of NAR on osteoclastogenesis, Bone marrow macrophages (BMMs) were exposed to NAR (0, 25, 50, 100 μM) during osteoclast formation. As shown in Figure 2C, the control group formed many TRAP-positive multinucleated osteoclasts. Osteoclast formation was inhibited by NAR (Figure 2D,E).

### 2.2. F-Actin Ring Formation

**Figure 3:**
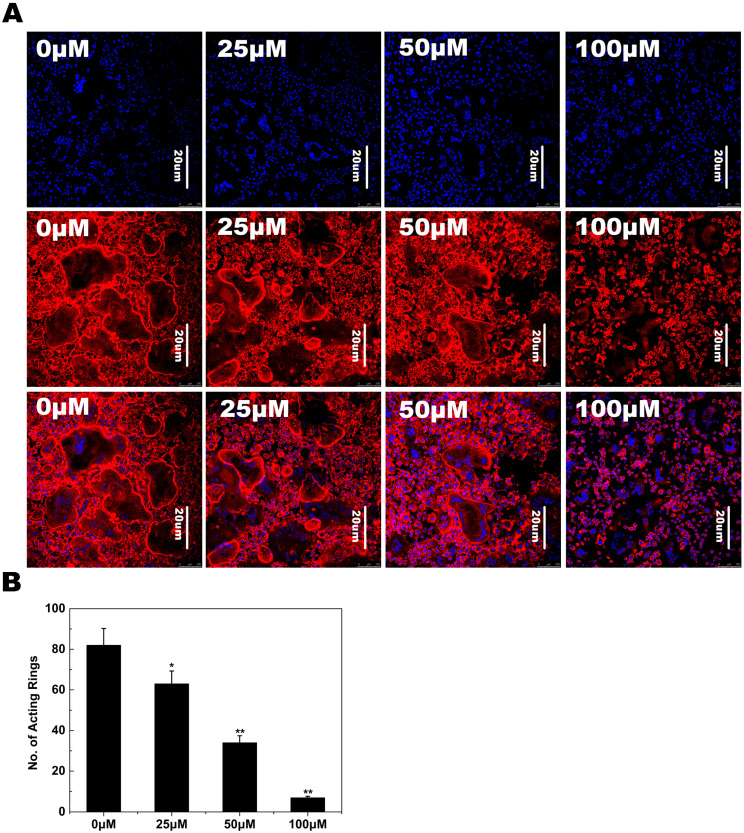
Naringenin (NAR) inhibited receptor activator of the nuclear factor-κB ligand (RANKL)-induced F-actin ring formation. (**A**) Bone marrow macrophages (BMMs) were incubated with macrophage colony-stimulating factor (M-CSF) (30 ng/mL) and RANKL (50 ng/mL), followed by treatment with vehicle or NAR. Cells were fixed and stained for F-actin; (**B**) The number of osteoclasts with F-actin rings. * *p* < 0.05; ** *p* < 0.01.

The effects of NAR on osteoclastogenesis were further studied by examining RANKL-induced osteoclast F-actin ring formation, a readily observable characteristic of mature osteoclasts and a prerequisite for osteoclast bone resorption during osteoclastogenesis. Confocal microscopy revealed F-actin ring formation and characteristic podosomal condensation in control osteoclasts. However, the size and number of F-actin rings were significantly decreased in cells incubated with NAR (Figure 3A), suggesting that NAR suppressed F-actin ring formation.

### 2.3. Osteoclast Bone Resorption

**Figure 4:**
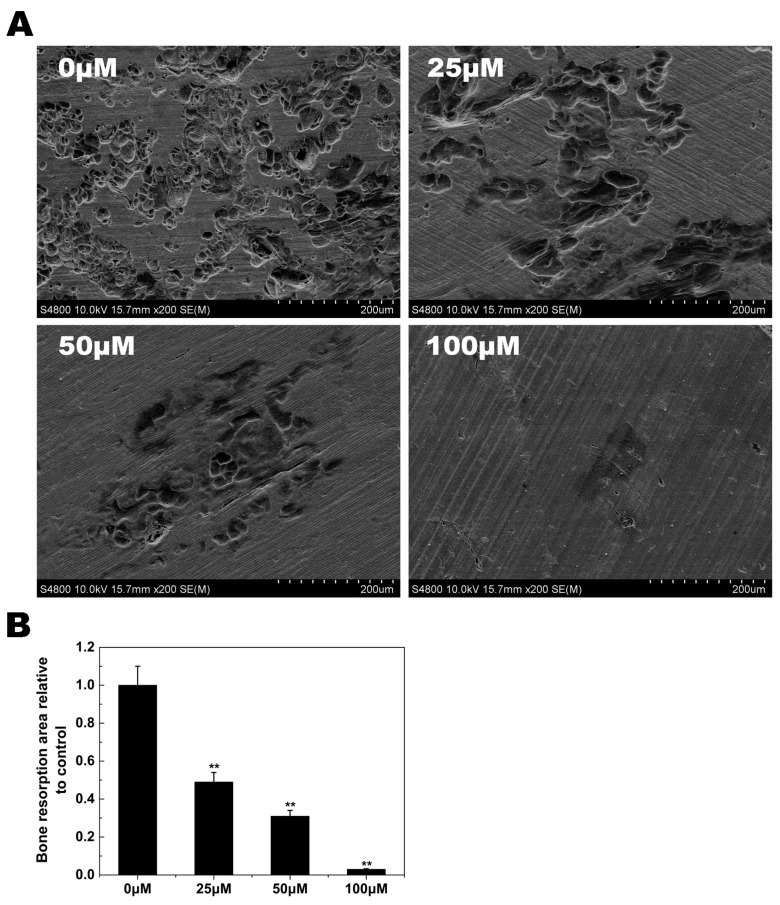
Naringenin (NAR) inhibited osteoclast-mediated bone resorption. Resorption pit areas were measured using ImageJ software (ImageJ software, Bethesda, MD, USA). All experiments were performed at least three times. (**A**) Representative scanning electron microscopy images of bone resorption pits; (**B**) The bone resorption area, relative to control. ** *p* < 0.01.

Since the formation of a well-polarized F-actin ring is an essential prerequisite for efficient bone resorption by osteoclasts, we inferred from the previous experiments that osteoclast bone resorption would also be inhibited by NAR. As shown in Figure 4A, numerous bone resorption pits were observed on the surface of control bone slices. In bone slices exposed to NAR, the resorption area was decreased. These findings demonstrated that NAR impaired osteoclast bone resorption *in vitro*.

### 2.4. RANKL-Induced Gene Expression

**Figure 5:**
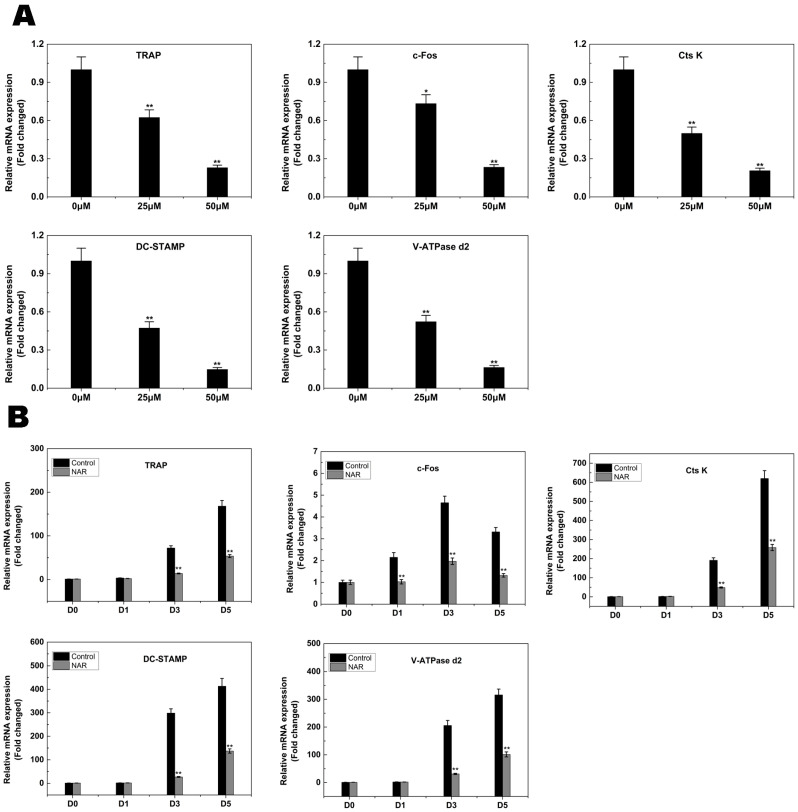
Naringenin (NAR) suppressed receptor activator of the nuclear factor-κB ligand RANKL-induced expression of osteoclast-specific genes. Bone marrow macrophages (BMMs) were cultured with macrophage colony stimulating factor (M-CSF; 30 ng/mL) and RANKL (50 ng/mL), with or without NAR. The expression of *c-Fos*, *TRAP*, cathepsin K (*CtsK*), dendritic cell-specific trans membrane protein (*DC-STAMP*) and vacuolar-type H^+^-ATPase domain 2 (*V-ATPase d2*) was analyzed by real-time quantitative polymerase chain reaction and the results were normalized to the expression of glyceraldehyde-3-phosphate dehydrogenase (*GAPDH*). (**A**) Levels of the indicated mRNAs following exposure to NAR (0, 25, or 50 μM); (**B**) Levels of the indicated mRNAs following exposure to 50 μM NAR for 0, 1, 3, or 5 days. All experiments were performed at least three times. * *p* < 0.05; ** *p* < 0.01.

The expression levels of several specific genes are upregulated during osteoclast differentiation. We utilized qPCR to examine and quantify the RANKL-induced mRNA expression of osteoclast-related genes including *NFATc1*, *TRAP*, *V-ATPase d2*, *CtsK*, *DC-STAMP*, and *c-Fos*. The results showed that the expression of these genes was inhibited by NAR in a concentration- and time-dependent manner (Figure 5A,B).

### 2.5. RANKL-Induced p38 Signaling and Anisomycin on NAR-Treated Osteoclastogenesis

**Figure 6:**
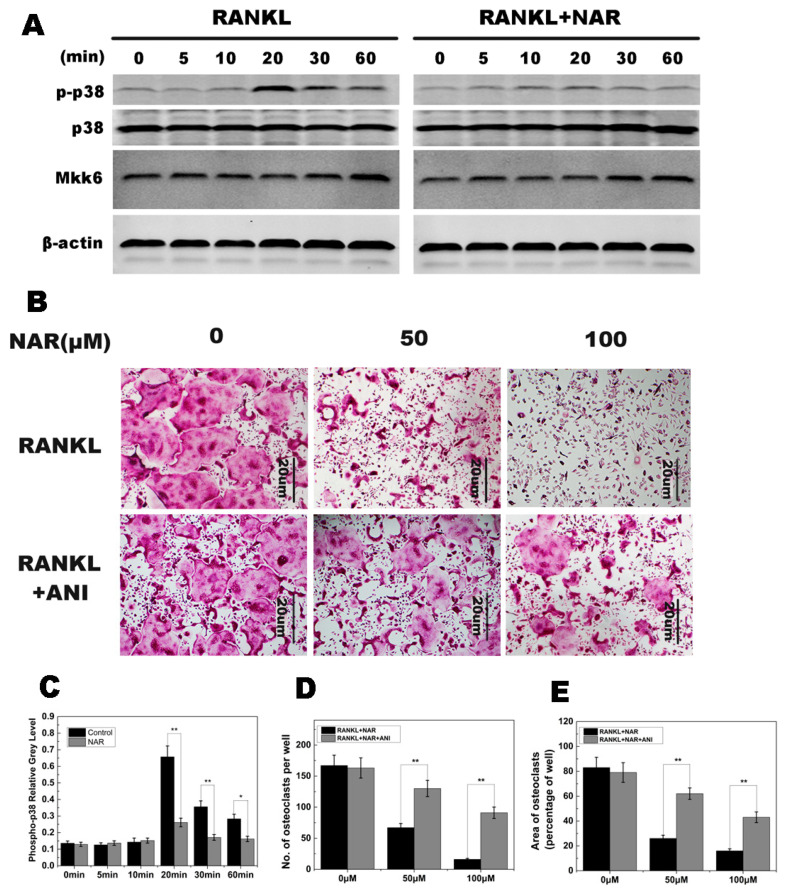
Naringenin (NAR)-mediated suppression of receptor activator of the nuclear factor-κB ligand (RANKL)-induced effects on p38 signaling. (**A**) p38 phosphorylation was increased within 20–60 min of stimulation with RANKL in the control group and greatly reduced by treatment with 200 μM NAR. MAPK kinase 6 (MKK6) was not affected by 200 μM NAR. (**B**) Anisomycin (ANI) counteracted the inhibitory effect of NAR on osteoclast formation. BMMs were stimulated with 30 ng/mL macrophage colony-stimulating factor (M-CSF), 50 ng/mL RANKL, and the indicated concentrations of NAR. In the rescue groups, cells were also treated with 2.5 ng/mL anisomycin. All treated cells were stained for tartrate-resistant acid phosphatase (TRAP). (**C**) The gray levels corresponding to p38 phosphorylation were quantified and normalized relative to β-actin by using ImageJ software Version 1.46, and the results presented in this graph confirmed the data presented in (**A**). (**D**) The number of TRAP-positive cells. (**E**) The area occupied by TRAP-positive cells. * *p* < 0.05; ** *p* < 0.01.

In this study, p38 phosphorylation was increased within 20–60 min of stimulation with RANKL in the control group. However, p38 phosphorylation was greatly reduced by exposure to NAR (Figure 6A). Quantitative analysis confirmed these results (Figure 6C). Further studies showed that MKK6 was not affected by NAR treatment (Figure 6A). These results suggested that NAR inhibited phosphorylation of p38 signaling during osteoclast differentiation.

As described above, osteoclast formation was inhibited by NAR, which may be due to reduced p38 phosphorylation. To confirm these observations, following the treatment with NAR, BMM cells were treated with anisomycin, which has been reported to activate p38 pathways. Osteoclast formation was inhibited in cells treated with NAR. However, in cells also treated with anisomycin, the impaired osteoclastogenesis was restored and mature osteoclasts were observed (Figure 6B).

### 2.6. RANKL-Induced NF-κB, ERK, JNK Signaling

**Figure 7:**
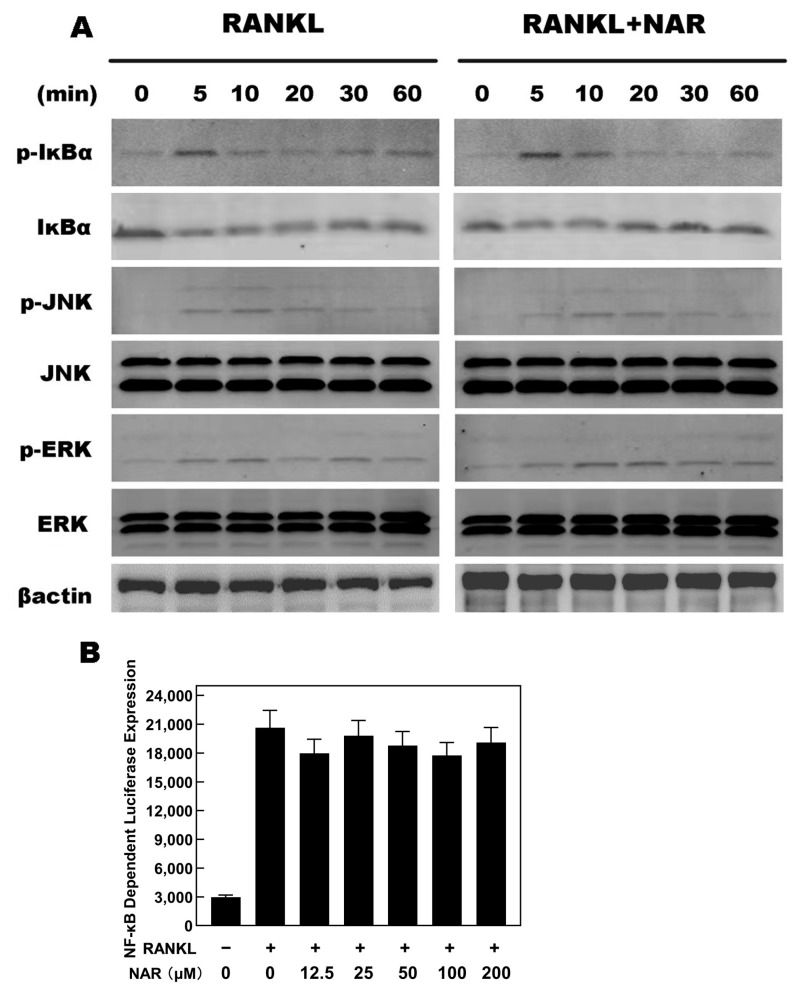
Naringenin (NAR)-mediated effects on receptor activator of the nuclear factor-κB ligand RANKL-induced nuclear factor κB (NF-κB), c-Jun *N*-terminal kinase (JNK), and extracellular signal-regulated kinase (ERK) signaling. (**A**) The levels of p-IκBα, IκBα, p-JNK and p-ERK were unaffected by exposure to 200 μM NAR. (**B**) Luciferase reporter assays showed that NAR did not affect RANKL-induced NF-κB signaling.

We used western blots and luciferase assays to investigate the NF-κB, ERK, and JNK signaling pathways. Similar levels of IκBα and p-IκBα, were observed in control and NAR groups (Figure 7A). This observation was supported by luciferase reporter gene assays (Figure 7B). p-ERK and p-JNK levels were also similar in control and NAR groups (Figure 7A). These data indicated that NAR inhibited osteoclastogenesis without affecting the NF-κB, ERK, and JNK signaling pathways.

### 2.7. RANKL-Induced NFATc1 Expression

**Figure 8:**
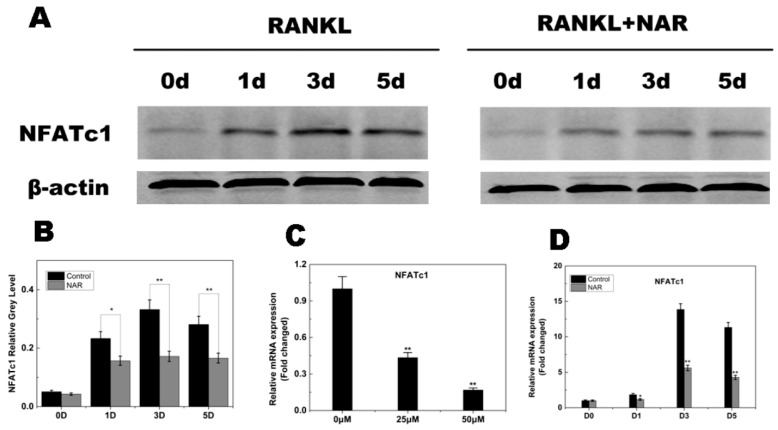
Naringenin (NAR)-mediated suppression of receptor activator of the nuclear factor-κB ligand (RANKL)-induced nuclear factor of activated T cells c1 (NFATc1) signaling. (**A**) Suppression of RANKL-induced NFATc1 signaling by NAR (200 μM). (**B**) Quantitative analysis of *NFATc1* expression. (**C**) Levels of the indicated NFATc1 mRNAs following exposure to NAR (0, 25 or 50 μM). (**D**) Levels of the indicated NFATc1 mRNAs following exposure to 50 μM NAR for 0, 1, 3 or 5 days. * *p* < 0.05; ** *p* < 0.01.

NFATc1 is a master regulator of osteoclastogenesis and osteoclast function. Therefore, we investigated the effects of NAR on RANKL-induced *NFATc1* expression. The data presented in Figure 8C,D indicated that *NFATc1* transcriptional activity increased when the cells were stimulated by RANKL. NAR inhibited this activity in a concentration- and time-dependent manner. To confirm the effect of NAR on *NFATc1* expression, we examined the NFATc1 protein level using western blot analysis. NFATc1 protein levels increased when cells were exposed to RANKL, and NAR attenuated this increase (Figure 8A,B), suggesting that NAR suppressed RANKL-induced *NFATc1* expression.

### 2.8. Titanium Particle-Induced Osteolysis

**Figure 9:**
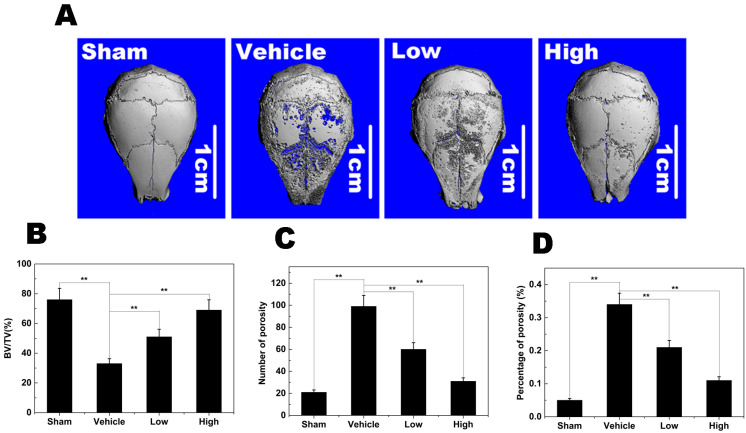
Naringenin (NAR) attenuated titanium (Ti) particle-induced mouse calvarial osteolysis. (**A**) Representative micro-computed tomography (CT) three-dimensional images from each group; (**B**) bone volume/tissue volume ratio (BV/TV); (**C**) number of pores; (**D**) the percent porosity in each sample. ** *p* < 0.01.

To explore the effects of NAR on pathological osteolysis, we used a mouse calvaria model of Ti particle-induced osteolysis. The degree of particle-induced osteolysis was assessed using high-resolution micro-CT and histological examination. Compared with the sham group (no Ti particles), the vehicle group (administration of Ti particles in PBS) showed significant calvarial osteolysis. When NAR (10 mg/kg, low; 25 mg/kg, high) was administered with the Ti particles, osteolytic bone loss was reduced (Figure 9A). Quantitative analysis demonstrated that the high and low concentrations of NAR significantly increased the bone volume/tissue volume (BV/TV) ratio (Figure 9B), and decreased the number of pores and the percent porosity in the ROI of the calvaria (Figure 9C,D).

**Figure 10:**
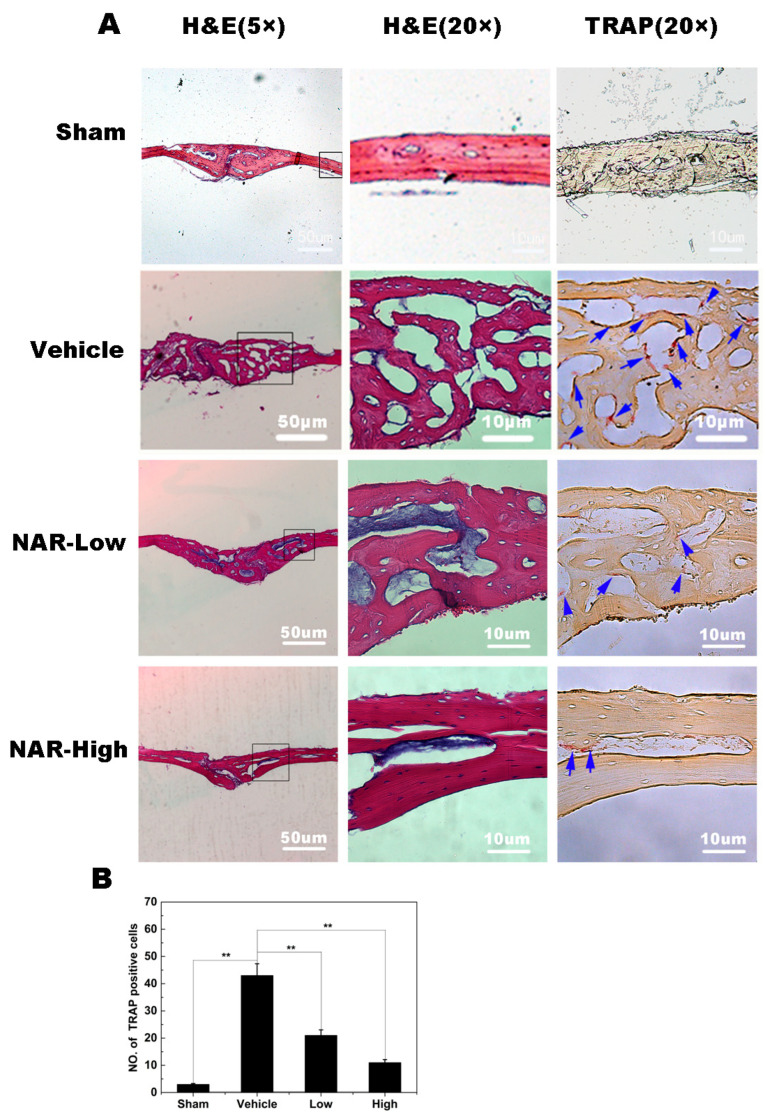
Histological and histomorphometric analysis of the effects of naringenin (NAR) on titanium (Ti) particle-induced mouse calvarial osteolysis. (**A**) Representative histological sections stained with hematoxylin and eosin (H&E) and stained for tartrate-resistant acid phosphatase (TRAP). (**B**) Histomorphometric analysis of the number of TRAP-positive multinucleated osteoclasts in the sections. ** *p* < 0.01.

Histological analysis confirmed the attenuation of Ti particle-induced bone erosion by NAR (Figure 10A). TRAP staining (Figure 10A; blue arrowheads), revealed numerous osteoclasts along the eroded bone surface in the vehicle (Ti in PBS) group. The erosion surface was reduced and the number of osteoclasts decreased in the NAR treatment groups (Figure 10B). These data were consistent with the micro-CT results, indicating that NAR may be an effective anti-resorptive agent for the reduction of Ti particle-induced osteolysis.

### 2.9. Schematic Diagram Describing the Mechanism by Which Naringenin (NAR) Inhibits Osteoclast Differentiation and Function

**Figure 11:**
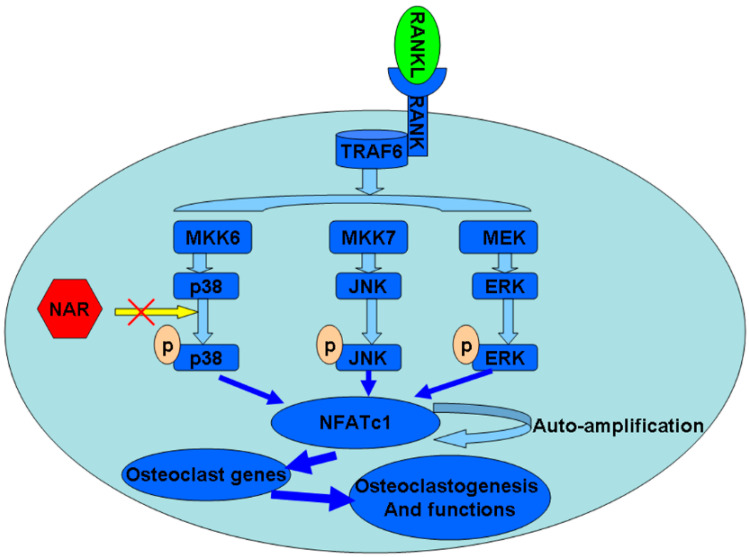
Schematic diagram describing the mechanism by which Naringenin (NAR) inhibits osteoclast differentiation and function.

Activation of RANK by its ligand leads to the expression of osteoclast-specific genes during differentiation and the activation of resorption by mature osteoclasts. The subsequent activation of p38, JNK, and ERK is essential for osteoclast differentiation, survival, and activation. MKK6, MKK7, and MEK are upstream of p38, JNK, and ERK, respectively. Activated p38, JNK, and ERK then stimulate transcription factors such as NFATc1. NFATc1 regulates the expression of genes associated with osteoclast differentiation and function. NAR attenuated p38 phosphorylation and decreased the expression of NFATc1 and its downstream genes. Therefore, NAR blocks osteoclast differentiation and bone resorption (Figure 11).

## 3. Discussion

TJA is frequently used to treat severe joint disease. Although instability, infection, pain and periprosthetic bone fractures are common reasons for reoperation in the first five years after the initial surgery, the most frequent cause of late failure is aseptic loosening accompanied by pathological osteolysis induced by wear particles [31]. Pathological bone destruction occurs when osteoclasts are present in elevated numbers and/or exhibit enhanced activity, leading to excessive bone resorption. Diseases associated with excessive bone resorption include periprosthetic osteolysis, osteoporosis, rheumatoid arthritis, multiple myeloma, and metastatic cancers [31,32,33,34]. During the last two decades, many advances have been made in the treatment of osteolytic diseases, but treatment options are still far from ideal. Therefore, attempts to develop better treatment options are being pursued.

In this study, we demonstrated that NAR inhibited RANKL-induced osteoclastogenesis *in vitro* and attenuated titanium particle-induced osteolysis *in vivo*.

Osteolysis during aseptic loosening is most likely due to increased bone resorption by osteoclasts [35]. Therefore, osteoclasts are considered as the viable target for therapeutic agents to treat wear particle-induced osteolysis [4]. *In vitro*, we demonstrated that osteoclast differentiation and formation were inhibited, F-actin ring formation was impaired, and the number and area of bone resorption pits were dramatically decreased after NAR treatment.

RANKL and its cognate receptor RANK have been characterized as essential factors for osteoclastogenesis [36]. Docking of RANKL to its receptor, RANK, rapidly activates at least five distinct signaling cascades mediated by protein kinases during osteoclastogenesis and activation. These five signaling cascades include IκBα, JNK, p38, ERK, and Src pathways [37]. Activated MAPK then led to the stimulation of transcription factors such as NFATc1 [13,38,39], a master regulator of osteoclast differentiation [40,41,42]. Attenuation of phosphorylation statuses of p38 leads to the inhibition of transcription factor-NFATc1 at the protein level [43,44]. The over-expression of *NFATc1* accelerates RANKL-induced osteoclast differentiation, and transduced *NFATc1* increased osteoclast formation even without stimulation by RANKL. In addition, NFATc1-deficent embryonic stem cells fail to differentiate into osteoclasts even in the presence of RANKL [40]. Using western blot analysis, we demonstrated that NAR inhibited RANKL-induced p38 signaling. MKK6, an upstream protein of p38, was not affected by NAR treatment.

When anisomycin, an activator of p38 [45,46], was used to treat BMMs, after treatment with NAR, impaired osteoclastogenesis was restored. We also demonstrated that two other MAPK signaling pathways, JNK and ERK, were not affected. NF-κB was also unaffected, as confirmed by a NF-κB luciferase reporter gene assay.

We confirmed that NAR inhibited *NFATc1* transcriptional activity and expression at the protein and mRNA levels. We examined the expression of NFATc1-regulated genes, such as *TRAP*, *cathepsin K*, *V-ATPase d2*, and found that they were all down-regulated by NAR, suggesting that NAR affects not only the expression of *NFATc1* but also the expression of the downstream genes.

Furthermore, we demonstrated that NAR significantly reduced titanium particle-induced osteolysis *in vivo*. Through micro-computed tomography (CT) three-dimensional images from each group, we found that obvious osteolysis was present in the Ti group to a much greater extent than in the sham group, whereas NAR treatment suppressed particle-induced osteolysis in a dose-dependent manner, with no observed adverse effects. These results were further supported by histological analysis of tissues stained with hematoxylin and eosin (H&E) and stained for TRAP. The sham sections exhibited few inflammatory and osteolytic changes. The vehicle group showed an obvious inflammatory reaction and prominent osteolysis, whereas the NAR-treated groups exhibited reduced inflammation and osteolysis. The number of TRAP-positive multinucleated osteoclasts on the section slices of NAR-treated groups was decreased drastically compared to the vehicle group.

However, there are some limitations of this study. First, side effect of Ti particles is another issue we should consider. Previous studies have indicated that metal implants undergo corrosion inside the human body by various mechanisms [47]. Metallic ions are released into the tissue surrounding metal implants, as well as distant organs (liver, spleen, and lymph nodes) and body fluids (serum and urine) [48]. Exposed to metal ions, T-lymphocytes proliferate and differentiate to produce excessive inflammatory cytokines, such as IL-1, IL-6, and TNF-α that will activate osteoclastogenesis and trigger a series of systemic immune response [49]. Here, we observed no major side effects on the mice in our experiments. Secondly, in consideration of generating osteolysis model, ultra-high molecular weight polyethylene (UHMWPE) is of paramount concern to today’s joint replacement surgeons because significantly more polyethylene particles (70%–90%) appear in tissues around metal-on-polyethylene prostheses [50,51]. However, metal particles, though relatively fewer in the tissue, are also an important factor contributing to osteolysis [52,53,54]. Ti particle and UHMWPE would ultimately activate osteoclast formation and function to induce osteolysis *in vivo*. Therefore, both animal models could be suitable for investigating the effect of NAR on osteolysis [42,52]. In our following related studies, we would like to establish the osteolytic animal model with the use of UHMWPE particles.

Taken together, our results demonstrated the inhibitory effects of NAR on osteoclastogenesis and osteoclast function *in vitro* and *in vivo*. We also showed that NAR suppressed the p38 signaling pathway. Our data strongly suggested that NAR could be developed as a treatment for osteoclast-mediated resporptive diseases including periprosthetic osteolysis.

## 4. Experimental Section

### 4.1. Media, Reagents, and Cells

Alpha modification of Eagle medium (α-MEM) and fetal bovine serum (FBS) were obtained from Gibco-BRL (Sydney, Australia). The cell counting kit (CCK-8) was purchased from Dojindo (Kumamoto, Japan). Soluble human recombinant macrophage-colony stimulating factor (M-CSF) and bacteria-derived recombinant mouse RANKL were supplied by R&D (R&D Systems, Minneapolis, MN, USA). Naringenin (NAR) was purchased from Sigma–Aldrich (St. Louis, MO, USA) and dissolved in dimethyl sulfoxide and then diluted to appropriate concentrations in culture medium. Specific antibodies against p38, phosphorylated-p38 (Thr180/Tyr182), ERK, phosphorylated-ERK (Thr202/Tyr204) (p-ERK), JNK, phosphorylated-JNK (Thr183/Tyr185) (p-JNK), inhibitor of nuclear factor kappa-B kinase subunit alpha (IκBα), phospho-IκBα, MAPK kinase 6 (MKK6), nuclear factor of activated T cells c1 (NFATc1), and β-actin were obtained from Cell Signaling Technology (Cambridge, MA, USA). RAW264.7 cells were purchased from the American Type Culture Collection (Rockville, MD, USA).

### 4.2. Cytotoxicity Assay

The cytotoxic effects of NAR were determined using a CCK-8 assay. BMMs were seeded in 96-well plates at a density of 8 × 10^3^ cells/well, and cultured in complete α-MEM supplemented with 30 ng/mL M-CSF for 24 h. Cells were then treated with different concentrations of NAR (0, 3.125, 6.25, 12.5, 25, 50, 100, 200, 400, 800 μM) for 48 h. CCK-8 buffer (10 μL) was added to each well, and cells were incubated at 37 °C for another 2 h. Then the absorbance was measured at a wavelength of 450 nm (650 nm reference) with an ELX800 absorbance microplate reader (Bio-Tek, Winooski, VT, USA). The half-maximal inhibitory concentration (IC_50_) value was calculated using GraphPad Prism software version 5.0c (San Diego, CA, USA).

### 4.3. BMMs Isolation and Osteoclasts Culture

For primary cultures, BMMs were obtained from femurs and tibias of 6-week-old C57/BL6 mice. Cells were cultured in a T75 flask in α-MEM supplemented with 10% FBS, 1% penicillin/streptomycin, and 30 ng/mL M-CSF for 24 h. Floating cells were removed, and the adherent cells were cultured at 37 °C, in 5% CO_2_ for another 3 days until the cells were fully confluent. The BMMs were then plated into a 96-well plate at a density of 8 × 10^3^ cells/well in complete α-MEM supplemented with 30 ng/mL M-CSF, 50 ng/mL RANKL, and different concentrations of NAR (0, 25, 50, 100 μM). Culture media were replaced every 2 days until mature osteoclasts had formed. Cells were then washed twice with PBS, fixed with 4% paraformaldehyde for 20 min. Osteoclasts were identified by positive staining for TRAP. TRAP-positive cells with more than three nuclei were counted under a microscope.

### 4.4. F-Actin Ring Immunofluorescence

To measure F-actin ring immunofluorescent staining, osteoclasts were fixed with 4% paraformaldehyde for 15 min at room temperature (RT) and permeabilized for 5 min with 0.1% *v*/*v* Triton X-100. Cells were incubated with rhodamine-conjugated phalloidin (1:100, Invitrogen; Carlsbad, CA, USA) diluted in 0.2% *w*/*v* BSA–PBS for 1 h at RT and washed extensively with 0.2% *w*/*v* BSA–PBS and PBS. Cells were then incubated with Hoechst 3342 dye (1:5000; Invitrogen) for visualization of nuclei, washed with PBS and mounted with ProLong Gold anti-fade mounting medium (Invitrogen). Fluorescence was measure with the NIKON A1Si spectral detector confocal system equipped with 20× (dry) lenses (Nikon, Tokyo, Japan). Fluorescence images were collected using the systems NIS-C Elements software (Nikon, Tokyo, Japan) and analysed using ImageJ software (ImageJ software, Bethesda, MD, USA).

### 4.5. Bone Resorption Pit Assay

In order to measure bone resorption, BMMs were seeded on bone slices in 96-well plates at a density of 8 × 10^3^ cells/well with three replicates and incubated with M-CSF (30 ng/mL), RANKL (50 ng/mL) and NAR (0, 25, 50, 100 μM). Cells that had adhered to bone slices were removed by mechanical agitation. Bone slice images were captured using field-emission scanning electron microscopy (FESEM, Hitachi S-4800, CamScan, Tokyo, Japan) at 10 kV. Three fields were randomly selected for each bone slice for further analysis. Pit areas were quantified using ImageJ software. Similar independent experiments were repeated at least three times.

### 4.6. RNA Extraction and Quantitative PCR Assay

Quantitative PCR (qPCR) was used to measure specific gene expression in osteoclasts. BMMs were plated in 6-well plates at a density of 1 × 10^5^ cells per well and cultured in complete α-MEM supplemented with 30 ng/mL M-CSF and 50 ng/mL RANKL. For the concentration-response assays, cells were incubated with NAR (0, 25, and 50 μM) for 5–7 days until mature osteoclasts formed. For the time dependence assay, cells were incubated with NAR (50 μM) for 0, 1, 3, or 5 days. Total RNA was extracted using the Qiagen RNeasy Mini kit (Qiagen, Valencia, CA, USA) and cDNA was synthesized from 1 μg of total RNA using reverse transcriptase (TaKaRa Biotechnology, Otsu, Japan). Real-time PCR was performed using the SYBR Premix Ex Tag kit (TaKaRa) and an ABI 7500 Sequencing Detection System (Applied Biosystems, Foster City, CA, USA). The cycling conditions were: 40 cycles of denaturation at 95 °C for 5 s and amplification at 60 °C for 24 s. Glyceraldehyde-3-phosphate dehydrogenase (*GAPDH*) was used as the standardization gene, and all reactions were run in triplicate. The mouse primer sequences of cathepsin K (*CtsK*), *TRAP*, dendritic cell-specific trans membrane protein (*DC-STAMP*), vacuolar-type H+-ATPase domain 2 (*V-ATPase d2*), *c-fos*, and *NFATc1*, *GAPDH* were as follows: *CtsK* forward: 5'-CTTCCAATACGTGCAGCAGA-3' and reverse: 5'-TCTTCAGGGCTTTCTCGTTC-3'; *TRAP* forward: 5'-CTGGAGTGCACGATGCCAGCGACA-3' and reverse: 5'-TCCGTGCTCGGCGATGGACCAGA-3'; *DC-STAMP* forward: 5'-AAAACCCTTGGGCTGTTCTT-3' and reverse: 5'-AATCATGGACGACTCCTTGG-3'; *V-ATPase d2* forward: 5'-AAGCCTTTGTTTGACGCTGT-3' and reverse: 5'-TTCGATGCCTCTGTGAGATG-3'; *c-Fos* forward: 5'-CCAGTCAAGAGCATCAGCAA-3' and reverse: 5'-AAGTAGTGCAGCCCGGAGTA-3'; *NFATc1* forward: 5'-CCGTTGCTTCCAGAAAATAACA-3' and reverse: 5'-TGTGGGATGTGAACTCGGAA-3'; GAPDH forward: 5'-ACCCAGAAGACTGTGGATGG-3' and reverse: 5'-CACATTGGGGGTAGGAACAC-3'.

### 4.7. NF-κB Luciferase Reporter Gene Assay

To investigate whether NAR affected nuclear factor κB (*NF-κB*) gene expression, RAW264.7 cells were stably transfected with a luciferase reporter construct (p-NF-κB-TA-Luc). Briefly, cells were plated in a 24-well plate at a density of 1 × 10^5^ cells per well and incubated for 24 h. Then cells were treated with different concentrations of NAR (0, 12.5, 25, 50, 100, and 200 μM) for 1 h, prior to incubation with 50 ng/mL RANKL for an additional 8 h. Cells were then lysed and luciferase activity was measured using the Promega Luciferase Assay System (Promega, Madison, WI, USA) following the manufacturer’s instructions.

### 4.8. Osteoclastogenesis Rescue Assay

BMMs were seeded into 96-well plates at a density of 8 × 10^3^ cells/well in complete α-MEM supplemented with 30 ng/mL M-CSF. After adhering to the well, cells were treated with 50 ng/mL RANKL and 0, 50, or 100 μM NAR. In addition, a rescue group was treated with 2.5 ng/mL anisomycin, a potent activator of p38 [45,46], following treatment with NAR. Cells were fixed and stained for TRAP as soon as mature osteoclasts had formed; the number and area of TRAP-positive cells were then determined.

### 4.9. Western Blot Analysis

RAW264.7 cells were seeded in 6-well plates at a density of 5 × 10^5^ cells per well. After pretreatment with vehicle or NAR (200 μM) for 4 h, the RAW264.7 cells were stimulated with RANKL for 0, 5, 10, 20, 30 or 60 min. Then RAW264.7 cells were then washed in PBS twice and lysed in radio-immunoprecipitation assay (RIPA) lysis buffer (50 mM Tris–HCl, 150 mM NaCl, 5 mM EDTA, 1% Triton X-100, 1 mM sodium fluoride, 1 mM sodium vanadate, 1% deoxycholate) supplemented with phenylmethylsulfonyl fluoride (The Lys Neng Bo Cai Corp., Shanghai, China). The lysates were centrifuged at 12,000 rpm for 10 min and supernatants were collected.

Protein concentrations were measured with a bicinchoninic acid (BCA) assay. Thirty microgram of each protein lysate was resolved using sodium dodecyl sulfate-polyacrylamide gel electrophoresis (SDS-PAGE) on 10% gels and transferred to polyvinylidene difluoride membranes (Millipore, Bedford, MA, USA). The membranes were blocked with 5% skim milk in TBS-Tween (TBS; 0.05 M Tris, 0.15 M NaCl pH 7.5, and 0.2% Tween-20) for 1 h, incubated with primary antibodies diluted in 1% skim milk powder in TBS–Tween overnight at 4 °C. Membranes were then washed and incubated with the appropriate secondary antibodies conjugated with IRDye 800CW (molecular weight 1162 Da). Antibody reactivity was detected by exposure in an Odyssey infrared imaging system (LI-COR, Lincoln, NE, USA).

### 4.10. Titanium Particle-Induced Calvarial Osteolysis Model

A wear particle-induced osteolysis model was created as previously reported [55]. All mice were housed at 20–26 °C and in 40%–70% humidity with 12 h light/dark cycles. All animal care and experimental procedures were approved by Animal Care Committee of Shanghai Jiao Tong University (Animal Ethics Approval #201350, approval date: 11 March 2013). To remove endotoxins adherent on titanium (Ti) particles, commercial pure Ti particles were sterilized by baking at 180 °C for 6 h, followed by treatment of 70% ethanol for 48 h. Twenty healthy 8-week-old C57BL/J6 mice were randomly assigned into four groups (5 mice in each group): sham phosphate buffered saline (PBS) control (sham), Ti particle osteolysis with PBS vehicle (vehicle), and Ti particle osteolysis with low NAR (10 mg/kg/day; low) and Ti particle osteolysis with high NAR (25 mg/kg/day; high). 2 days after the implantation, NAR or PBS was injected into the periosteum every other day for 14 days. To control any acute pain induced by the bone grafting, buprenorphine (0.5 mg/kg) was given post-operatively, and sterile PBS (0.1 mL) containing 1:100 penicillin/streptomycin were used for antibiotic prophylaxis. No adverse effects or mortality occurred during the experiment. At the end of the experiment, the mice were sacrificed, and the calvaria were excised and fixed in 4% paraformaldehyde for micro-CT (computerized tomography) analysis.

### 4.11. Micro-Computed Tomography (micro-CT) Scanning

A high-resolution micro-CT scanner (Skyscan 1176; Skyscan; Aartselaar, Belgium) was used for qualitative and quantitative analyses of osteolysis in mouse calvaria at a resolution of 9 mm using the following settings: X-ray voltage, 50 kV; electric current, 500 mA; rotation step, 0.7°. To reduce metal artifacts, the wear particles were removed before scanning. After removal of the particles, a square region of interest (ROI) around the midline suture was chosen for further qualitative and quantitative analysis. Bone volume-tissue volume ratio (BV/TV), the number of porosity (number of pores), and percent porosity for each sample were measured in the ROI.

### 4.12. Histological and Histomorphometric Analysis

After micro CT scanning, the calvarial samples were decalcified in 10% EDTA for 6–8 weeks, and then embedded in paraffin. Histological sections were prepared for tartrate-resistant acid phosphatase (TRAP) and H&E staining. The specimens were then examined and photographed under a high quality microscope. The number of TRAP-positive multinucleated osteoclasts was counted in each sample.

### 4.13. Statistical Analysis

The data have been expressed as mean ± SD. The results were analyzed using the AVONA and Post ad Hoc tests with the SPSS 13.0 software (SPSS Inc., Chicago, IL, USA). *p* < 0.05 indicated a significant difference between groups.

## 5. Conclusions

NAR exerts protective effects *in vitro* mainly by inhibiting osteoclastogenesis through the p38 signaling pathway. Moreover, we demonstrated that NAR protects against Ti-particle-induced osteolysis *in vivo*. Our data strongly suggested that NAR could be developed as a treatment for osteoclast-mediated resporptive diseases including periprosthetic osteolysis.
