# Correction: Wang et al. The Inhibition of RANKL-Induced Osteoclastogenesis through the Suppression of p38 Signaling Pathway by Naringenin and Attenuation of Titanium-Particle-Induced Osteolysis. *Int. J. Mol. Sci*. 2014, *15*, 21913–21934

**DOI:** 10.3390/ijms26125426

**Published:** 2025-06-06

**Authors:** Wengang Wang, Chuanlong Wu, Bo Tian, Xuqiang Liu, Zanjing Zhai, Xinhua Qu, Chuan Jiang, Zhengxiao Ouyang, Yuanqing Mao, Tingting Tang, An Qin, Zhenan Zhu

**Affiliations:** 1 Shanghai Key Laboratory of Orthopedic Implants, Department of Orthopedics, Shanghai Ninth People’s Hospital, Shanghai Jiao Tong University School of Medicine, Shanghai 200011, China; wwg1532@163.com (W.W.); challengewu1988@163.com (C.W.); poetian@163.com (B.T.); shliuxuqiang@163.com (X.L.); eryijiuyuan@163.com (Z.Z.); xinhua_qu@126.com (X.Q.); jc720u@hotmail.com (C.J.); terryoy@126.com (Z.O.); 13761360486@163.com (Y.M.); wzy1532@sina.com (T.T.); 2 Department of Orthopedics, Hunan Provincial Tumor Hospital and Tumor Hospital of Xiangya School of Medicine, Central South University, Changsha 410013, China

In the original publication [1], there was an error in Figures 6B, 7A, 8A and 10A as published. The authors sincerely apologize for this oversight and have confirmed that these errors resulted from the inadvertent use of images. The corrected versions of Figure 6, Figure 7, Figure 8 and Figure 10 are provided below.

The authors state that the scientific conclusions are unaffected. This correction was approved by the Academic Editor. The original publication has also been updated.

## Figures and Tables

**Figure 6:**
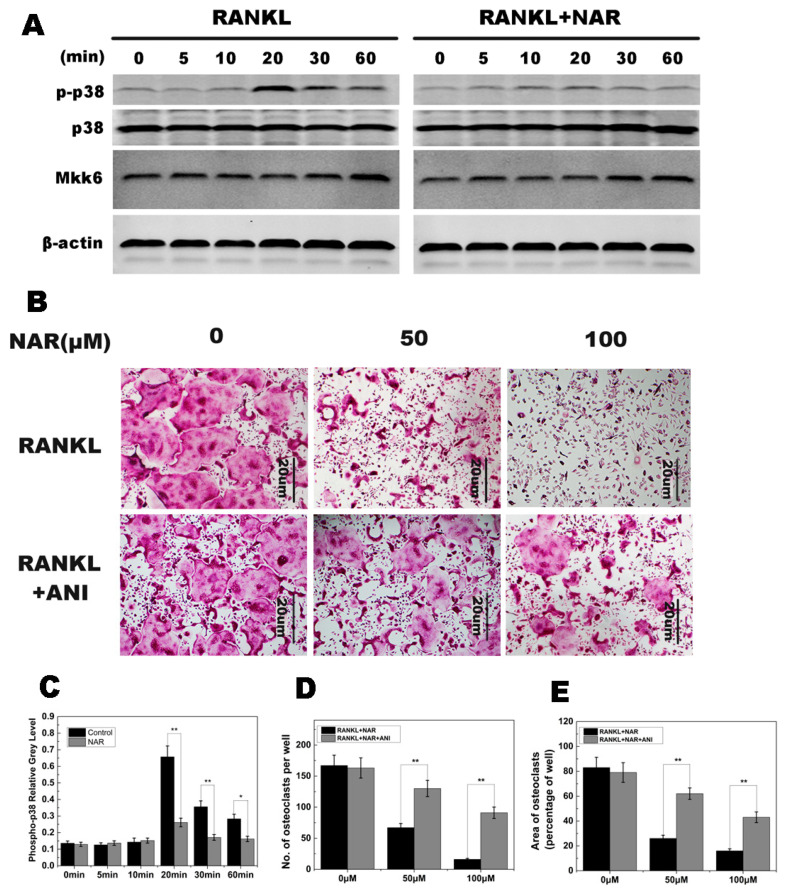
Naringenin (NAR)-mediated suppression of receptor activator of the nuclear factor-κB ligand (RANKL)-induced effects on p38 signaling. (**A**) p38 phosphorylation was increased within 20–60 min of stimulation with RANKL in the control group and greatly reduced by treatment with 200 μM NAR. MAPK kinase 6 (MKK6) was not affected by 200 μM NAR. (**B**) Anisomycin (ANI) counteracted the inhibitory effect of NAR on osteoclast formation. BMMs were stimulated with 30 ng/mL macrophage colony-stimulating factor (M-CSF), 50 ng/mL RANKL, and the indicated concentrations of NAR. In the rescue groups, cells were also treated with 2.5 ng/mL anisomycin. All treated cells were stained for tartrate-resistant acid phosphatase (TRAP). (**C**) The gray levels corresponding to p38 phosphorylation were quantified and normalized relative to β-actin by using ImageJ software Version 1.46, and the results presented in this graph confirmed the data presented in (**A**). (**D**) The number of TRAP-positive cells. (**E**) The area occupied by TRAP-positive cells. * *p* < 0.05; ** *p* < 0.01.

**Figure 7:**
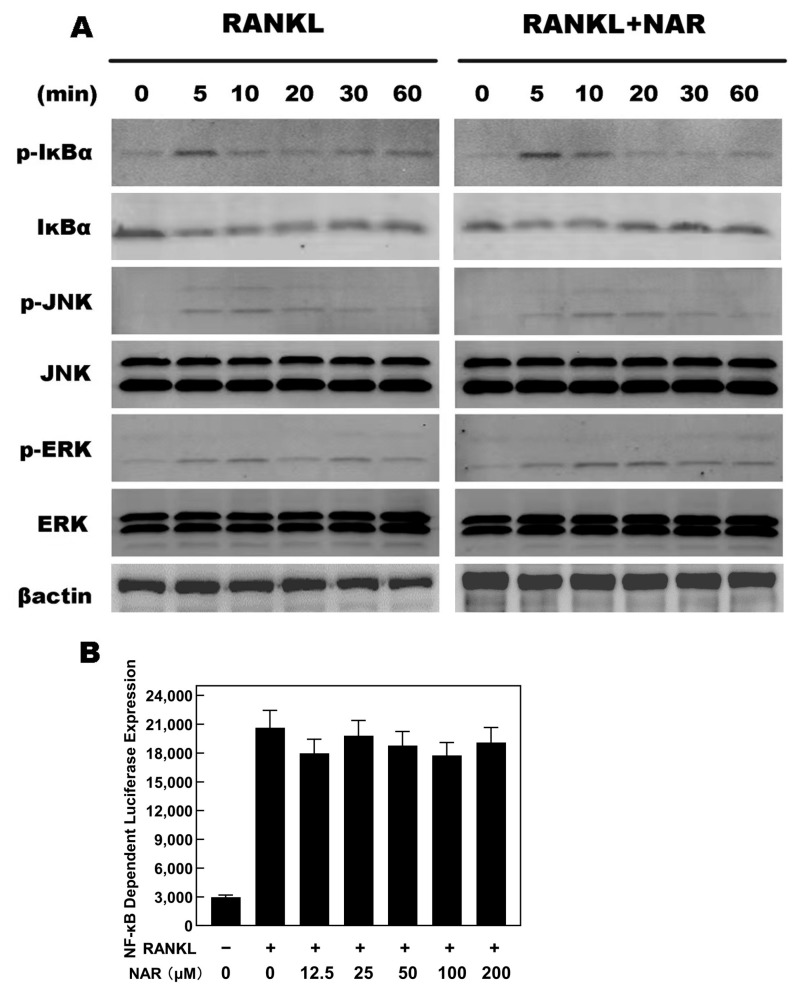
Naringenin (NAR)-mediated effects on receptor activator of the nuclear factor-κB ligand RANKL-induced nuclear factor κB (NF-κB), c-Jun N-terminal kinase (JNK), and extracellular signal-regulated kinase (ERK) signaling. (**A**) The levels of p-IκBα, IκBα, p-JNK and p-ERK were unaffected by exposure to 200 μM NAR. (**B**) Luciferase reporter assays showed that NAR did not affect RANKL-induced NF-κB signaling.

**Figure 8:**
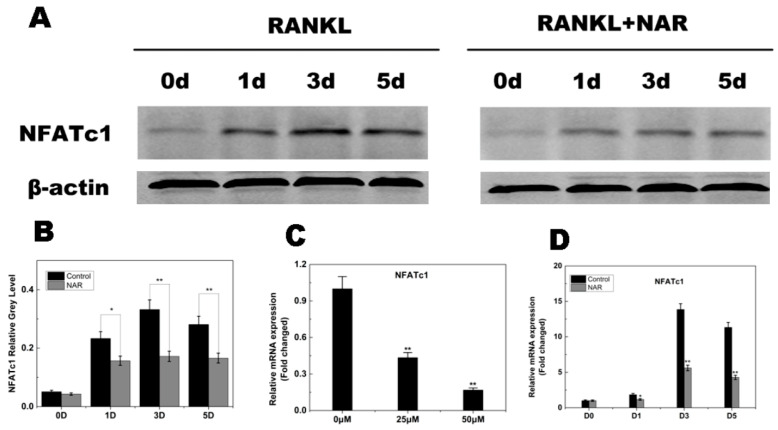
Naringenin (NAR)-mediated suppression of receptor activator of the nuclear factor-κB ligand (RANKL)-induced nuclear factor of activated T cells c1 (NFATc1) signaling. (**A**) Suppression of RANKL-induced NFATc1 signaling by NAR (200 μM). (**B**) Quantitative analysis of NFATc1 expression. (**C**) Levels of the indicated NFATc1 mRNAs following exposure to NAR (0, 25 or 50 μM). (**D**) Levels of the indicated NFATc1 mRNAs following exposure to 50 μM NAR for 0, 1, 3 or 5 days. * *p* < 0.05; ** *p* < 0.01.

**Figure 10:**
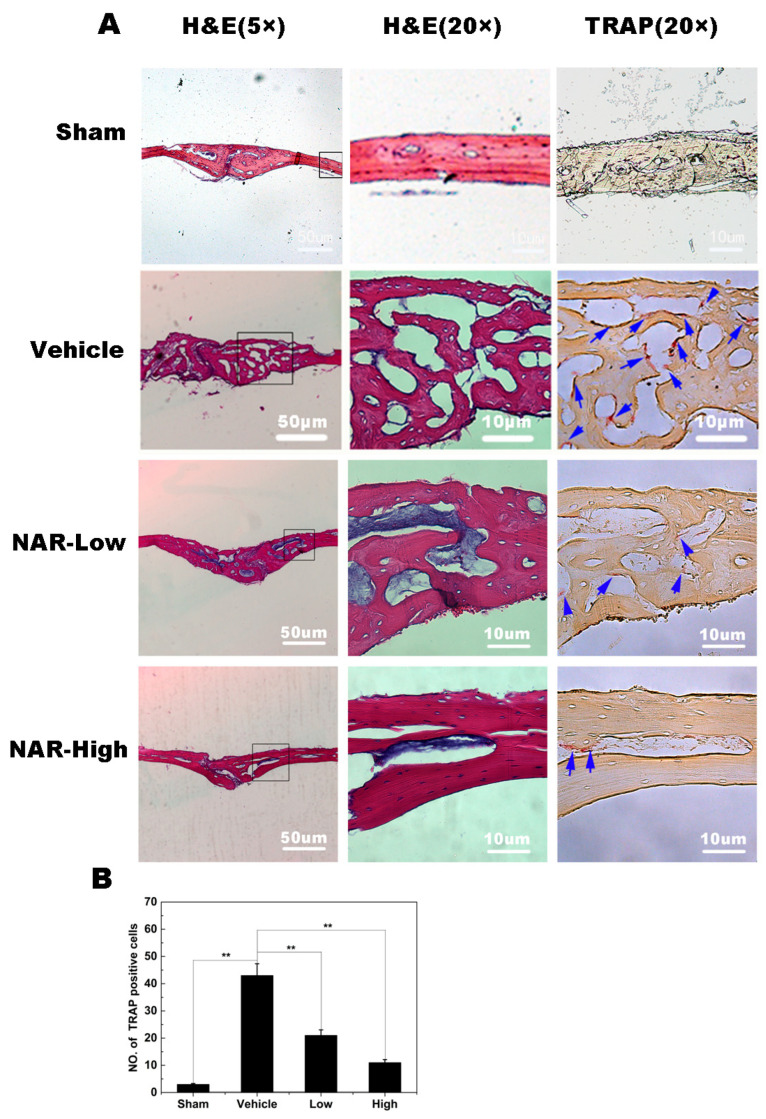
Histological and histomorphometric analysis of the effects of naringenin (NAR) on titanium (Ti) particle-induced mouse calvarial osteolysis. (**A**) Representative histological sections stained with hematoxylin and eosin (H&E) and stained for tartrate-resistant acid phosphatase (TRAP). (**B**) Histomorphometric analysis of the number of TRAP-positive multinucleated osteoclasts in the sections. ** *p* < 0.01.

## References

[1] Wang W., Wu C., Tian B., Liu X., Zhai Z., Qu X., Jiang C., Ouyang Z., Mao Y., Tang T. (2014). The Inhibition of RANKL-Induced Osteoclastogenesis through the Suppression of p38 Signaling Pathway by Naringenin and Attenuation of Titanium-Particle-Induced Osteolysis. Int. J. Mol. Sci..

